# Analysis of Relationship between the Body Mass Composition and Physical Activity with Body Posture in Children

**DOI:** 10.1155/2016/1851670

**Published:** 2016-09-28

**Authors:** Justyna Wyszyńska, Justyna Podgórska-Bednarz, Justyna Drzał-Grabiec, Maciej Rachwał, Joanna Baran, Ewelina Czenczek-Lewandowska, Justyna Leszczak, Artur Mazur

**Affiliations:** Medical Faculty, University of Rzeszów, 26 Warszawska Street, 35-205 Rzeszów, Poland

## Abstract

*Introduction*. Excessive body mass in turn may contribute to the development of many health disorders including disorders of musculoskeletal system, which still develops intensively at that time.* Aim*. The aim of this study was to assess the relationship between children's body mass composition and body posture. The relationship between physical activity level of children and the parameters characterizing their posture was also evaluated.* Material and Methods*. 120 school age children between 11 and 13 years were enrolled in the study, including 61 girls and 59 boys. Each study participant had the posture evaluated with the photogrammetric method using the projection moiré phenomenon. Moreover, body mass composition and the level of physical activity were evaluated.* Results*. Children with the lowest content of muscle tissue showed the highest difference in the height of the inferior angles of the scapulas in the coronal plane. Children with excessive body fat had less slope of the thoracic-lumbar spine, greater difference in the depth of the inferior angles of the scapula, and greater angle of the shoulder line. The individuals with higher level of physical activity have a smaller angle of body inclination.* Conclusion*. The content of muscle tissue, adipose tissue, and physical activity level determines the variability of the parameter characterizing the body posture.

## 1. Introduction

Increasing the percentage of children with postural problems requires focus on prevention and analysis of existing postural problems [1]. Postural problems in school-age children are one of the most common health problems in this population. Some postural problems are typical of human growth and development, while others are harmful and can affect the quality of life negatively [2]. Most postural problems start in childhood. The body posture depends on many factors, including age, gender, race, somatic structure of the bone-joints and muscles, mental status, lifestyle, and sport [3].

One of the main factors differentiating posture is age. A child's body posture differs from the adult one. Moreover, differences in the body posture in children of different age groups are noted [4]. Less obvious is the diversity of posture in terms of gender, because gender related diversity of posture is not specific to every age group and affects mainly young people in the second critical period of posturogenesis [5].

It is worth emphasizing that both overweight and obesity [6, 7] as well as low level of physical activity and sedentary lifestyle have a significant impact on the postural parameters in many children [3, 8]. Excessive body mass in obese or overweight children may cause a decrease in the stability and the need to seek postural mechanisms of adaptation. This can cause changes in habitual balance axis resulting in increased lumbar lordosis of abdominal protrusion and pelvic anteversion. Over time, excessive shortening or lengthening may occur, which in combination with pelvic anteversion leads to internal rotation of the hip joint and initiation of valgus knees and flat feet [9]. Also, other researchers point to the excessive abdominal protrusion, increased lumbar lordosis, severe valgus knees, and flat feet in children with excess body mass [10, 11].

Early onset of these problems is the result of early development of excessive body mass, starting in preschool children. According to World Health Organization, over 40 million children worldwide under the age of five were overweight in 2011 [12] and in 2013 more than 42 million children suffered from overweight or obesity. If the situation does not change, the current generation of children will probably live shorter than their parents [13]. Due to the increase in the prevalence of overweight and obesity among children and adolescents it can be assumed that the number of individuals with postural problems will increase in the coming years.

Cross-sectional and longitudinal evaluations showed that the deterioration of posture, even in children with normal weight, is associated with increasing adiposity [14, 15]. It has been demonstrated that children with normal body mass index (BMI) may have a high content of fat in the body, especially around the viscera with less muscle mass [16]. Individuals with normal weight who were diagnosed with metabolic disorders are referred to as metabolically obese normal weight (MONW) [17, 18]. There are few reports describing the relationship between the composition of body mass and body posture in children. It has not been examined whether there is a relationship between the percentages of adipose or muscle tissue with body posture parameters. The present paper concentrates on this issue.

## 2. Paper's Purpose

The aim of this study was to assess the relationship between children's body mass composition and body posture. The relationship between physical activity level of children and the parameters characterizing their posture was also evaluated.

## 3. Material and Method

The study included 120 primary school students aged 11–13 years (61 girls and 59 boys). The schools participating in the study were randomly chosen.

The study was conducted after obtaining written consent from the schools headmasters, the participating children's parents, and the children themselves. The study was approved by the local Bioethics Committee.

The study took place at nursing clinics in selected educational institutions. To ensure reliability of measurement, children with mobility disorders, neurological deficits, orthopedic disease, and those unable to keep their balance in standing or with the aid of orthopedic equipment were excluded from the study.

### 3.1. Photogrammetric Method

Photogrammetric method using the projection moiré phenomenon (MORA 4 Generation System) was used in order to assess selected parameters characterizing the posture. Scientific research confirms that the results obtained by means of photogrammetric method are very similar to the X-ray outcomes [19]. According to Saad et al., the photogrammetric measures are replicable and can be used as a complementary test to reduce the number of X-ray examinations necessary to monitor the spine [20]. In subsequent studies on the reliability of photogrammetric method, Saad et al. found a strong correlation between the evaluators and the test-retest analyzes [21]. Another team of researchers also confirmed the accuracy of photogrammetric method in the evaluation of body posture [22].

The study was conducted according to generally accepted principles provided by the manufacturer. In order for a test procedure to be reliable and reproducible, examinations were carried out at the same time of a day (morning hours), using the same test equipment. The assessments were performed by a physiotherapist with 10 years of experience and extensive practice in photogrammetric measurements.

Each study participant was asked to remove their clothing from the waist up and stand in a fixed place, at a distance of 2.6 meter from the camera of the device. The measurement was conducted after a child adopted a relaxed position, a habitual posture.

**Figure 1:**
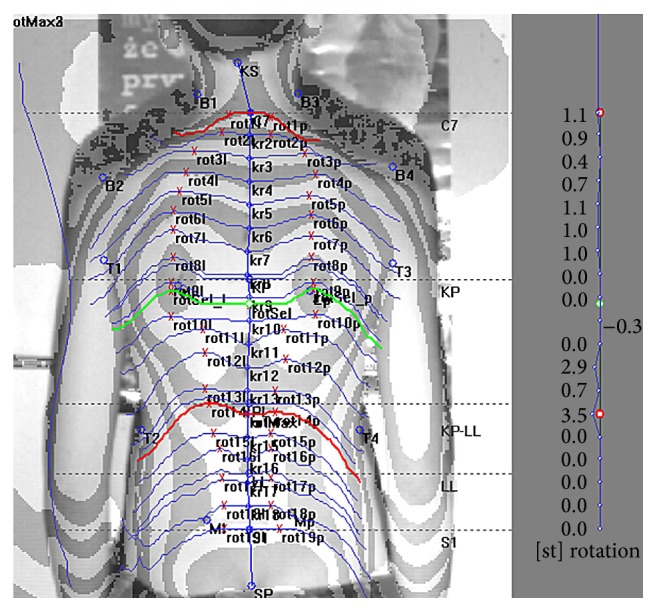
Photogrammetric survey sample. Source: own study. The authors obtained the student's consent to publish the image.

Before measurements were taken, the appropriate anthropometric points were marked on the back of each subject. The anthropometric points were determined by palpation and were marked with a dermatograph. The photogrammetric image was recorded after marking all the essential points and positioning the child with his or her back to the camera. Based on the marked points, the computer defined the parameters describing the body posture. Photogrammetric survey sample is presented in Figure 1.

**Figure 2:**
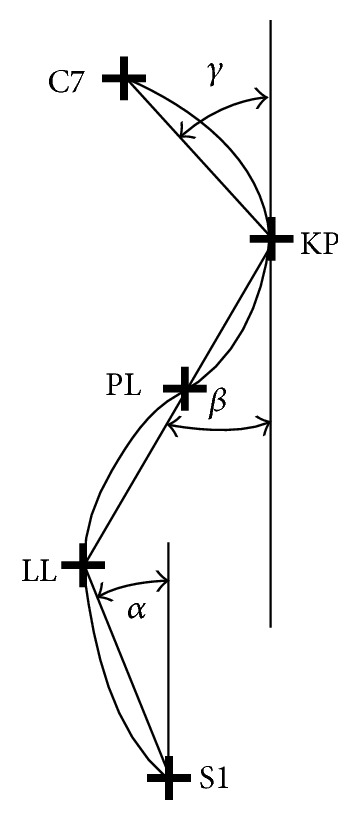
S1: transition of lumbar lordosis into the sacrum; LL: lumbar lordosis apex; PL: transition of kyphosis into lordosis; KP: thoracic kyphosis apex; C7: spinous process of the seventh cervical vertebra [23].

14 selected postural parameters were assessed in every child:

 ALPHA (°): slope of the lumbar spine.
 BETA (°): slope of the thoracic-lumbar spine.
 GAMMA (°): slope of the upper thoracic spine.
 KPT (°): angle of body inclination, specifying the forward and backward inclination of the body.
 KKP (°): angle of thoracic kyphosis [KKP = 180 − (BETA + GAMMA)] (Figure 2).
 GKP (mm): depth of thoracic kyphosis.
 KLL (°): angle of lumbar lordosis [KLL = 180 − (ALPHA + BETA)] (Figure 2).
 GLL (mm): depth of lumbar lordosis.
 KNT (°): the angle of trunk declination, determining the vertical decline of the C7-S1 line in the frontal plane (right, left).
 KLB (°): angle of the shoulder line.
 UL (mm): the difference in the height of the inferior angles of the scapulas in the coronal plane (inclination).
 UB (mm): the difference in the depth of the inferior angles of the scapulas (torsion).
 OL (mm): the difference in distance of the inferior angles of the scapulas from the spine.
 UK (mm): the maximum deviation of the line of the spinous processes from the C1–S1 line on the *x*-axis.

### 3.2. Anthropometric Measurements and Bioelectrical Impedance

The body height was measured to the nearest 0.1 cm using a portable stadiometer PORTSTAND 210. The body mass was determined to the nearest 0.1 kg. The measurements were performed under standard conditions, with subjects dressed in underwear, assuming upright and straight body posture, and bare feet.

Body mass index (BMI) was calculated by dividing the average body mass (kg) of each individual by his or her average squared height (m^2^). BMI values were transferred to suitable ranges of percentile bands. The applied centile grids for gender and age were developed in the framework of the Polish project entitled OLAF [24]. Based on the obtained percentile ranking, BMI status was classified into the following four categories: obese (BMI ≥ 95th percentile), overweight (BMI ≥ 85th percentile and < 95th percentile), healthy weight (BMI < 85th percentile and ≥ 5th percentile), and underweight (BMI < 5th percentile) [25].

Foot-to-foot bioelectrical impedance analysis (BIA) has been used to estimate body composition. The body composition analyzer (BC-420, Tanita) has been used. BIA method consists of measuring the impedance (electrical resistance, which consists of resistance and reactance) of tissue through which electrical current of low intensity is passed (<1 mA) [26]. BIA was performed in the early morning after an overnight fasting for at least 8 hours, because food or beverage consumption may decrease impedance by 4–15 Ω over a 2–4-hour period after meals, representing an error smaller than 3% [27].

Differences in the parameters characterizing posture depending on the percentage of muscle tissue were assessed. For this purpose, children were divided into 3 groups:

- Group I: a quarter of the lowest measurements (children with the lowest % of muscle tissue content).
- Group II: a half of the typical measurements (between the upper and lower quartile).
- Group III: a quarter of the highest measurements (children with the highest % of the muscle tissue content).

We also assessed whether there are differences in the parameters characterizing posture, depending on the percentage of body fat. In one of the largest studies on the percentage of fat and risk factors, it has been reported that excessive body fat levels (≥25% in boys and ≥30% in girls) were associated with greater health risk of cardiovascular diseases, diabetes, and other metabolic diseases [28]. Therefore, two groups of children were distinguished:

- Group I: healthy body fat percentage (<25% and <30% of body fat in boys and girls, resp.).
- Group II: excessive body fat percentage (≥25% and ≥30% of body fat in boys and girls, resp.).

### 3.3. Physical Activity

Physical activity of the children was assessed using* Physical Activity Questionnaire for Children* (PAQ-C). The questionnaire includes questions on physical activity undertaken in last 7 days. Each question is scored according to a five-point scale (1–5), where “1” is the lowest and “5” is the highest level of physical activity. Final result is the average value of the scored points; higher scores correspond to a higher level of physical activity [29]. The children were classified into two groups based of the PAQ score:

- Group I: low activity level, if PAQ for a given subject did not exceed the average of the entire population.
- Group II: high activity level, if PAQ for a given subject was higher than the average.

The differences between variables were verified using Mann-Whitney and Kruskal-Wallis tests, adopting the significance level at *p* < .05. Calculations were performed by means of SPSS software. In this study, the basic descriptive statistics applied for all tested parameters were mean [*x*] and standard deviation [SD].

## 4. Results

General characteristics of the children are presented in Table 1. The mean age of the children was 12.09 years (SD = 0.83) [girls 12.07 years (SD 0.85) versus boys 12.12 years (SD = 0.81)].

The mean body height in the group was 152.5 cm (SD = 9.52). The average body mass in the children was 49.63 kg (SD = 31.14). It was found that the average body mass was significantly higher in boys than girls (54.56 kg versus 44.85 kg, *p* = .049).

Mean BMI in the study group was 19.9 kg/m^2^ (SD = 3.55) and ranged from 13.5 to 31.2 kg/m^2^. The majority of examined children (*n* = 85) had a healthy weight, 25 was overweight, and 10 were obese.

The average percentage of body fat was 20.2% (SD = 7.28). The girls had statistically significant (*p* = .006) higher percentage of body fat compared to boys (22.2% versus 18.13%). Average body fat mass was at 10.11 kg (SD = 5.60) in the tested children. Excess body fat had 25 girls and 19 boys.

The average muscle mass in the children was 34.9 kg (SD = 7.45). Statistically significant (*p* < .001) higher muscle mass was found in boys than girls (37.45 kg versus 32.43 kg). The average percentage of muscle in the children was 74.91% (SD = 9.21). A higher percentage of muscle was found in boys than girls (76.06% versus 73.79%). This result was also statistically significant (*p* = .026).

The overall level of physical activity measured with PAQ-C in girls and boys was at a similar level (2.77 points versus 2.86 points).

The studies have shown that girls and boys differed in three out of 14 analyzed postural parameters (Table 2). It was found that the depth of thoracic kyphosis (GKP) in girls was significantly greater than boys (10.01 versus 6.50, *p* = .033). In boys significantly higher scores were found in the angle of shoulder line parameter KLB (−4.17); this value in girls amounted to −1.03, *p* = .009. This means asymmetry in shoulder setting (left shoulder was higher than the right one) which was more severe among boys than girls. In addition, statistically significant differences in the values of UL parameter between girls and boys (2.22 versus 2.43, *p* < .001) were found. This means that the inferior angle of the right scapula in girls is higher than in the left one and vice versa in boys: inferior angle of the left scapula is higher than in the right one.

The analysis of differences in the parameters characterizing posture showed statistically significant differences (*p* = .048) in the values of UL parameter between the groups of children, depending on the percentage of muscle mass. UL standard value in children with the least content of muscle tissue (between I and III quartiles) was 2.25. In children from Group II (moderate content of muscle tissue) it was negative (−1.59), and in children with the highest content of muscle it was an average of 0.66. This means that children with the highest content of muscle tissue show the smallest difference in the arrangements of the lower angles of the scapulas (the inferior angle of the right scapula is higher than the inferior angle of the left scapula by 0.66 mm). In contrast, children with the lowest content of muscle tissue are characterized by the biggest difference in the arrangement of the inferior angles of the scapulas (the inferior angle of the right scapula is higher than the inferior angle of the left scapula by 2.25 mm) (Table 3).

The results of the postural parameters analyses concerning the percentage of body fat are shown in Table 4. We observed that BETA value in subjects with healthy body fat was higher than in those with excessive fat content (6.96 versus 5.20, *p* = .020). We also found statistically significant differences in the angle of the shoulder line (KLB) between children with healthy and excessive fat content (−1.41 versus −4.58, *p* = .038). A similar pattern was detected in case of UB parameter. Children with healthy body fat obtained higher scores than those with excessive fat content (−2.77 versus −4.92, *p* = .040).

Analysis of the incidence of differences in the postural parameters, depending on the level of physical activity, showed one statistically significant relationship (Table 5). Children with a high level of physical activity were characterized by smaller inclination of the body (KTP) as compared to those with low levels of physical activity (6.66 versus −11.97, *p* = .035).

## 5. Discussion

Childhood obesity is a risk factor for several dysfunctions and diseases, with negative effects on morphology of the locomotor system, plantar pressure, and body stability. According to the World Health Organization, there are currently 42 million overweight and obese infants and young children worldwide. If this trend continues, by 2025 this number will have increased to 70 million. The increase rate of overweight and obese children is 30% higher in low- and middle-income countries than in developed countries [13].

Recent studies have shown that adipose tissue is not only an energy storage organ but also metabolically active endocrine organ producing proinflammatory cytokines (i.e., interleukin-6, TNF-*α*, and leptin) which take part in the formation of atherosclerotic plaque. It leads to chronic inflammation (as in obese individuals) and the development of metabolic disorders such as insulin resistance, carbohydrate and lipid disorders, and problems with coagulation. Excessive accumulation of body fat already in childhood exposes the body to a series of adverse changes in adulthood [30, 31].

Scarce evidence for the correlation between the composition of body mass and body posture of children can be found in the available literature. However, there are reports of the relationship between body mass composition and foot construction. Butterworth et al. analyzed the literature on the relationship of body composition and the structure and function of the foot. Evidence indicates that obesity is strongly associated with flat feet and valgus feet. The dynamic foot function is deteriorated, and plantar pressure is increased during walking. However, there is little evidence of the impact of isolated components of body mass, such as fat mass, the structure of the feet, and their function [32]. da Rocha et al. research also confirmed that obese children experience higher plantar pressure and have lower sensitivity than nonobese [33].

The results of the study on the analysis of the differences in posture between the sexes are inconclusive. Numerous reports indicate no statistically significant differences in the body posture of girls and boys. Barczyk et al. found no statistically significant differences between the parameters describing the posture in the frontal plane in the group of boys and girls aged 7 to 9 years [34]. Olszewska and Trzcińska in their studies using photogrammetric method conducted on a group of 353 students aged 8–11 years stated no significant differences between girls and boys in the lumbosacral slope, the thoracolumbar slope, and the upper thoracic slope [35]. Coelho et al. assessed the posture of children aged 5 to 14 and also found no statistically significant differences in the parameters characterizing posture of girls and boys [36]. Similar results were obtained by Drzał-Grabiec et al., explaining that the lack of differentiation between a group of boys and girls in body posture may be associated with age (7–9 years), in which the diversity of postures related to sex has not yet occurred [37].

The results of another study aimed to assess the prevalence of postural disorders in children aged 12-13 years depending on the BMI and sex and showed a greater prevalence of lordotic posture in girls rather than in boys, but these correlations were not statistically significant [38]. In turn, the results of our study indicate that there are differences between girls and boys in terms of three parameters characterizing posture. The depth of thoracic kyphosis (GKP) was much greater in girls than in boys, while boys had greater asymmetries in two parameters: (1) angle of the shoulder line (KLB) and (2) the difference in the height of the inferior angles of the scapulas in the coronal plane (UL). Similar results were obtained by Medojević and Jakšić, who observed that the differences related to gender dimorphism appear as early as in children at the age from 9 to 10. The authors explain this phenomenon by the fact that girls enter puberty at the age when they are more prone to certain postural disorders. Differences in body posture of boys and girls are also between 11 and 13 years of age, a period when the girls finish going through puberty, while boys reach it at this point [5].

Our study showed that there are significant differences in body posture among the subjects with healthy body fat and those with excessive body fat. Studied children with excessive body fat were characterized by a lesser slope of the thoracolumbar spine (BETA) than children with healthy body fat (5.20 versus 6.96). Reducing this value indicates flattening of kyphosis in the lower section. Similarly Grabara and Pstrągowska reported flattening of the thoracic kyphosis and increasing lumbar lordosis in children with overweight and obesity [39]. Górniak et al. in their study evaluated the quality of body posture in rural boys with deficient and excessive body fat in comparison to their peers with healthy content of this tissue in the body. A studied group of 589 boys of 7–18 years showed that excess fat tissue promotes the formation of abnormal anterior-posterior spinal curvatures and its deficiency abnormalities in the frontal plane [40]. Burdukiewicz et al. also showed higher incidence of abnormal anterior-posterior curvatures of the spine in children with excess and deficiency of body fat [41]. Lordosis develops in many overweight or obese people due to the protruding stomach. The spine is trying to keep the body upright and that way develops arch at the lower back to hold the body upright.

Motka and Shah demonstrated that obese children had poorer abdominal muscles strength resulting in a protruding abdomen. The result was anterior displacement of the center of gravity which was associated with enlargement of lumbar lordosis and pelvic anteversion. The constant overload spinal curvatures lead to deterioration of posture and strength of the abdominal muscles and hips [42]. In turn, the results of Malepe et al. demonstrated lack of significant relationship between BMI and kyphosis as well as scoliosis. However, the authors found an inverse relationship between BMI and lordosis suggesting increasing risk of developing lordosis as BMI increases [43]. Fu et al. also found no association of BMI with kyphosis and scoliosis but observed a significant relationship between BMI and lordosis [44]. In turn, the results of Latalski et al. did not indicate the existence of a statistically significant relationship between the prevalence of obesity and the occurrence of postural defects among school age children [3].

Our results showed that children with excessive body fat in the body have a greater asymmetry (in the frontal plane) in the angle of left shoulder (KLB) than children with healthy body fat (−4.58 versus −1.41). Similar relationships were found for UB parameter. There is greater asymmetry in the depth of the inferior angles of the scapula (in the sagittal plane) in children with excessive body fat (torsion of left scapula was observed in these children). We also found the presence of statistically significant difference between the UL parameters in the subject with varying percentages of muscle in the body. The study showed that children with the highest content of muscle tissue were characterized by the smallest difference in the slope of the inferior angles of the scapula in the coronal plane. Reverse dependencies were found in children with the lowest percentage of muscle. The asymmetry of the scapulas can affect, among others, the center of gravity. Rykała et al. showed that only a small asymmetry of scapulas setting has no impact on the projection of the center of gravity as well as in those without asymmetry present [45].

Nery et al. obtained similar data to our research results. Based on studies conducted in Brazil among more than 1,300 children, they found the relationship between obesity and the occurrence of asymmetry of shoulders and scapulas as well as scalene muscles asymmetry. The researchers suggested that there is a need for preventive action in the field of health, weight control, and maintaining trunk balance [46].

Decreased physical activity is assumed one of the reasons for the excessive accumulation of adipose tissue leading to overweight or obesity. Our study demonstrated that the children with a high level of physical activity were characterized by smaller body inclination (KTP) in relation to children with a low level of physical activity. Similar results were obtained by Mucha et al. The authors showed that young people aged 14–16 characterized by increased physical activity have a more correct value of lumbar lordosis, the angle of the sacrum, the difference in the distance of the scapulas from the spine, and greater spinal ROM in the sagittal and frontal planes than their peers with average and low physical activity level [47]. McMaster et al. showed that physical inactivity is a predisposing factor for spinal deformity [8]. Barańska et al. in their study showed that, in children aged 12–18 years, reduction of motor skills measured with Eurofit test to the greatest extent was due to increased body weight and disturbances in body posture (scapulas asymmetries, flat feet, and protruding scapulas) [48]. So vicious circle occurs. The absence or insufficient amount of movement causes the deposition of fat that can lead to overweight or obesity, at the same time increased body mass causes problems in movement and discourages physical activity. That is why it is important to educate and encourage as much as possible to take various forms of physical activity, especially in children. Steinberg et al. confirmed benefits of activating children with the problem of excessive body mass. They proved that the weight management program for obese children improved their shape, stability, and the vestibular system, which reduced the likelihood of falls in the participants of the study [49]. Similarly, recent studies of Schwanke et al. showed that a special program of exercise in overweight children led to positive changes in body posture, increased strength, and flexibility of muscles [50].

Investigating the relationship between body mass and postural parameters in children is preliminary research in this topic. Our findings need to be continued. Research should be carried out large-scale, taking into account a larger number of children and youth. Depending on the results, concrete and credible proposals may be formulated that will contribute to improving the principles of posture prophylaxis.

## 6. Conclusions

The present study suggests that constituents of body mass have an impact on the diversity of postural parameters. The content of fat determines the variability in the case of BETA, KLB, and UB parameters. The content of muscle tissue determines variation in UL parameter. High level of physical activity correlates with lower tilt of the trunk in children.

## Figures and Tables

**Table 1:** Differences in anthropometric parameters, body composition, and physical activity in relation to sex.

Parameter	Girls		Boys		Total		*p*
	*x*	SD	*x*	SD	*x*	SD	
Body height [cm]	151.68	9.83	153.43	9.18	152.54	9.52	.361
*Body mass [kg]*	*44.85*	*11.73*	*54.56*	*42.41*	*49.63*	*31.14*	*.049* ^*∗*^
BMI	19.3	3.6	20.5	3.4	19.9	3.6	.051
cc BMI	58.51	31.93	65.17	29.17	61.78	30.66	.187
*Fat [%]*	*22.20*	*7.63*	*18.13*	*6.31*	*20.20*	*7.28*	*.006* ^*∗*^
Fat mass [kg]	10.66	5.93	9.54	5.23	10.11	5.60	.306
*Muscle [%]*	*73.79*	*7.19*	*76.06*	*10.86*	*74.91*	*9.21*	*.026* ^*∗*^
*Muscle mass [kg]*	*32.43*	*6.16*	*37.45*	*7.84*	*34.90*	*7.45*	*<.001* ^*∗*^
PAQ	2.77	0.93	2.94	0.85	2.86	0.89	.180

Fat [%]: body fat percentage; muscle [%]: percentage of muscle; BMI: body mass index; cc BMI: BMI percentile; PAQ: overall level of physical activity. ^*∗*^Statistically significant results.

**Table 2:** Differences in the values of postural parameters depending on sex.

Variable	Girls		Boys		Total		*p*
	*x*	SD	*x*	SD	*x*	SD	
ALPHA (°)	10.57	13.00	8.62	10.58	9.61	11.87	.243
BETA (°)	6.94	3.91	5.66	3.08	6.31	3.57	.082
GAMMA (°)	25.08	22.74	26.87	25.56	25.96	24.08	.299
KPT (°)	−9.28	15.27	−9.41	15.09	−9.34	15.12	.423
KKP (°)	148.30	23.81	148.13	25.64	148.22	24.62	.713
*GKP (mm)*	*10.01*	*9.06*	*6.40*	*8.47*	*8.24*	*8.92*	*.033* ^**∗**^
KLL (°)	168.26	16.48	169.20	16.61	168.72	16.48	.525
GLL (mm)	−10.88	8.04	−9.66	8.09	−10.28	8.05	.465
KNT (°)	−0.74	1.31	−1.06	1.38	−0.89	1.35	.268
*KLB (*°)	*−1.03*	*6.29*	*−4.17*	*7.37*	*−2.58*	*6.99*	*.009* ^**∗**^
*UL (mm)*	*2.22*	*5.51*	*−2.43*	*6.84*	*−0.07*	*6.60*	*<.001* ^**∗**^
UB (mm)	−2.66	5.45	−4.48	7.17	−3.56	6.39	.184
OL (mm)	−2.38	8.69	−0.89	9.37	−1.65	9.02	.256
UK (mm)	−3.60	5.68	−2.82	4.84	−3.22	5.28	.349

ALPHA: slope of the lumbar spine; BETA: slope of the thoracic-lumbar spine; GAMMA: slope of the upper thoracic spine; KPT: angle of body inclination; KKP: angle of thoracic kyphosis; GKP: depth of thoracic kyphosis; KLL: angle of lumbar lordosis; GLL: depth of lumbar lordosis; KNT: the angle of trunk declination, determining the vertical decline of the C7-S1 line in the frontal plane; KLB: angle of the shoulder line; UL: the difference in the height of the inferior angles of the scapulas in the coronal plane; UB: the difference in the depth of the inferior angles of the scapulas; OL: the difference in distance of the inferior angles of the scapulas from the spine; UK: the maximum deviation of the line of the spinous processes from the C1–S1 line on the *x*-axis; (mm): millimeter; (°): degree. ^*∗*^Statistically significant results.

**Table 3:** Differences in the postural parameters depending on the percentage of muscle tissue.

Variable	Percentage of muscle mass						Total		*p*
	Low (Group I)		Typical (Group II)		High (Group III)				
	*x*	SD	*x*	SD	*x*	SD	*x*	SD	
ALPHA (°)	11.50	12.77	9.89	13.72	7.18	4.65	9.61	11.87	.256
BETA (°)	5.46	4.02	6.53	3.64	6.74	2.86	6.31	3.57	.431
GAMMA (°)	25.14	22.85	26.32	24.86	26.07	24.48	25.96	24.08	.771
KPT (°)	−9.04	15.06	−8.67	15.91	−11.00	13.86	−9.34	15.12	.889
KKP (°)	150.09	23.62	147.79	25.04	147.22	25.48	148.22	24.62	.773
GKP (mm)	7.24	11.43	7.99	8.85	9.73	5.77	8.24	8.92	.715
KLL (°)	167.80	16.90	170.42	19.55	166.24	6.23	168.72	16.48	.777
GLL (mm)	−7.98	8.47	−11.26	8.95	−10.64	4.88	−10.28	8.05	.218
KNT (°)	−0.85	1.45	−0.88	1.22	−0.97	1.52	−0.89	1.35	.898
KLB (°)	−3.97	6.11	−3.06	7.13	−0.21	7.19	−2.58	6.99	.246
*UL (mm)*	*2.25*	*6.61*	*−1.59*	*7.16*	*0.66*	*4.45*	*−0.07*	*6.60*	*.048* ^**∗**^
UB (mm)	−5.04	4.88	−3.60	6.87	−1.99	6.57	−3.56	6.39	.076
OL (mm)	0.10	8.75	−2.06	9.62	−2.56	8.06	−1.65	9.02	.516
UK (mm)	−3.82	5.45	−3.37	5.30	−2.30	5.11	−3.22	5.28	.324

ALPHA: slope of the lumbar spine; BETA: slope of the thoracic-lumbar spine; GAMMA: slope of the upper thoracic spine; KPT: angle of body inclination; KKP: angle of thoracic kyphosis; GKP: depth of thoracic kyphosis; KLL: angle of lumbar lordosis; GLL: depth of lumbar lordosis; KNT: the angle of trunk declination, determining the vertical decline of the C7-S1 line in the frontal plane; KLB: angle of the shoulder line; UL: the difference in the height of the inferior angles of the scapulas in the coronal plane; UB: the difference in the depth of the inferior angles of the scapulas; OL: the difference in distance of the inferior angles of the scapulas from the spine; UK: the maximum deviation of the line of the spinous processes from the C1–S1 line on the *x*-axis; (mm): millimeter; (°): degree. ^*∗*^Statistically significant results.

**Table 4:** Differences in the postural parameters depending on the percentage of body fat.

Variable	Percentage of body fat						*p*
	Healthy (Group I)		Excessive (Group II)		Total		
	*x*	SD	*x*	SD	*x*	SD	
ALPHA (°)	8.17	9.21	12.11	15.22	9.61	11.87	.133
*BETA (*°)	*6.96*	*3.39*	*5.20*	*3.64*	*6.31*	*3.57*	*.020* ^**∗**^
GAMMA (°)	25.33	24.21	27.05	24.09	25.96	24.08	.267
KPT (°)	−9.09	14.59	−9.78	16.15	−9.34	15.12	.233
KKP (°)	148.16	24.80	148.33	24.60	148.22	24.62	.931
GKP (mm)	9.43	7.97	6.18	10.13	8.24	8.92	.115
KLL (°)	168.42	14.84	169.24	19.17	168.72	16.48	.717
GLL (mm)	−11.26	7.84	−8.59	8.23	−10.28	8.05	.068
KNT (°)	−0.92	1.33	−0.84	1.40	−0.89	1.35	.553
*KLB (*°)	*−1.41*	*6.74*	*−4.58*	*7.04*	*−2.58*	*6.99*	*.038* ^*∗*^
UL (mm)	−0.71	6.44	1.04	6.79	−0.07	6.60	.264
*UB (mm)*	*−2.77*	*6.42*	*−4.92*	*6.19*	*−3.56*	*6.39*	*.040* ^*∗*^
OL (mm)	−1.89	7.88	−1.23	10.80	−1.65	9.02	.558
UK (mm)	−2.55	5.31	−4.36	5.07	−3.22	5.28	.135

ALPHA: slope of the lumbar spine; BETA: slope of the thoracic-lumbar spine; GAMMA: slope of the upper thoracic spine; KPT: angle of body inclination; KKP: angle of thoracic kyphosis; GKP: depth of thoracic kyphosis; KLL: angle of lumbar lordosis; GLL: depth of lumbar lordosis; KNT: the angle of trunk declination, determining the vertical decline of the C7-S1 line in the frontal plane; KLB: angle of the shoulder line; UL: the difference in the height of the inferior angles of the scapulas in the coronal plane; UB: the difference in the depth of the inferior angles of the scapulas; OL: the difference in distance of the inferior angles of the scapulas from the spine; UK: the maximum deviation of the line of the spinous processes from the C1–S1 line on the *x*-axis; (mm): millimeter; (°): degree. ^*∗*^Statistically significant results.

**Table 5:** Differences in the postural parameters, depending on the level of physical activity.

Variable	Level of physical activity						*p*
	Low (Group I)		High (Group II)		Total		
	*x*	SD	*x*	SD	*x*	SD	
ALPHA (°)	7.81	4.95	11.48	16.02	9.61	11.87	.487
BETA (°)	6.20	3.46	6.42	3.71	6.31	3.57	.819
GAMMA (°)	26.68	24.70	25.22	23.61	25.96	24.08	.819
*KPT (*°)	*−11.97*	*14.08*	*−6.66*	*16.07*	*−9.34*	*15.12*	*.035* ^**∗**^
KKP (°)	147.54	25.24	148.93	24.16	148.22	24.62	.769
GKP (mm)	7.73	8.26	8.76	9.61	8.24	8.92	.539
KLL (°)	165.57	6.63	171.97	22.15	168.72	16.48	.404
GLL (mm)	−10.17	7.90	−10.40	8.27	−10.28	8.05	.981
KNT (°)	−0.97	1.56	−0.81	1.10	−0.89	1.35	.767
KLB (°)	−2.36	7.06	−2.79	6.98	−2.58	6.99	.924
UL (mm)	−0.23	6.97	0.10	6.24	−0.07	6.60	.949
UB (mm)	−3.01	6.68	−4.12	6.09	−3.56	6.39	.494
OL (mm)	−2.44	7.68	−0.83	10.23	−1.65	9.02	.456
UK (mm)	−2.92	5.55	−3.52	5.01	−3.22	5.28	.828

ALPHA: slope of the lumbar spine; BETA: slope of the thoracic-lumbar spine; GAMMA: slope of the upper thoracic spine; KPT: angle of body inclination; KKP: angle of thoracic kyphosis; GKP: depth of thoracic kyphosis; KLL: angle of lumbar lordosis; GLL: depth of lumbar lordosis; KNT: the angle of trunk declination, determining the vertical decline of the C7-S1 line in the frontal plane; KLB: angle of the shoulder line; UL: the difference in the height of the inferior angles of the scapulas in the coronal plane; UB: the difference in the depth of the inferior angles of the scapulas; OL: the difference in distance of the inferior angles of the scapulas from the spine; UK: the maximum deviation of the line of the spinous processes from the C1–S1 line on the *x*-axis; (mm): millimeter; (°): degree. ^*∗*^Statistically significant results.

## Additional Points

Lack of information about maturity levels can be recognized as a weakness of our study. The maturity level may affect body fat, particularly among girls aged 10–13. However, the results of one of the largest studies on the percent of fat in children and adolescents aged 5 to 18 years indicated that, regardless of the subjects' age and the level of puberty, body fat ≥25% in boys and ≥30% in girls contributes to greater health risk [28]. Therefore, we decided to adopt the above-mentioned cut-off points in our analyses.

Due to the difference in the level of maturation of children aged 10 to 13, we understand that there might be differences in body posture and body composition in these age groups and that the ideal would be to perform separate data analyses that could infer results for these populations individually.

The large sample size in this study allowed for assessment of relationship between children's body posture and body mass composition; however, it was not large enough to carry out logistic regression analysis. Therefore, it is essential to undertake further studies to confirm the current study findings in a larger sample size of children and adolescents at different age groups, as well in pathological populations (with defects of body posture). This will allow for stratification into subgroups according to age, allowing a more thorough investigation of the influence of gender on posture according to the age of each group.

Assessment of the level of physical activity performed by means of the self-assessment method (PAQ-C) can be considered one of the study limitations. However, the results of research indicate that the PAQ-C is a reliable and reproducible tool used to assess physical activity in children [29].

In spite of the limitations, we believe that the results of the study contributed with new information in the knowledge of the Public Health.
